# Hysteresis-Free and Bias-Stable Organic Transistors Fabricated by Dip-Coating with a Vertical-Phase-Separation Structure

**DOI:** 10.3390/ma17071465

**Published:** 2024-03-22

**Authors:** Bingxi Wang, Xiaowen Yin, Shuwen Yu, Haibo Wang

**Affiliations:** 1 Key Laboratory of Automobile Materials, Ministry of Education, School of Materials Science and Engineering, Jilin University, Changchun 130012, China; wangbingxi0228@163.com (B.W.);; 2 Division of Energy Research Resources, Dalian Institute of Chemical Physics, Chinese Academy of Sciences, Dalian 116023, China

**Keywords:** organic transistors, viscosity, dielectric polymer, vertical phase separation, carrier mobility, device stability

## Abstract

The morphology of organic films plays a pivotal role in determining the performance of transistor devices. While the dip-coating technique is capable of producing highly oriented organic films, it often encounters challenges such as limited coverage and the presence of defects in gaps between strips, adversely affecting device performance. In this study, we address these challenges by increasing solution viscosity through the incorporation of a substantial proportion of dielectric polymers, thereby enhancing the participation of additional molecules during the film formation process when pulled up. This method produces continuous and oriented organic films with a notable absence of gaps, significantly improving the carrier mobility of transistor devices by more than twofold. Importantly, the fabricated devices exhibit remarkable reliability, showing no hysteresis even after 200 cycles of measurement. Furthermore, the current and threshold voltages of the devices demonstrate exceptional stability, maintaining steady after 10,000 s of bias measurement. This approach provides a solution for the cost-effective and large-scale production of organic transistors, contributing significantly to the advancement of organic electronics.

## 1. Introduction

Organic semiconductors have exhibited many promising features, including intrinsic flexibility, being lightweight, efficient large-scale processing capabilities, etc. [1,2]. In particular, solution-processing techniques provide a versatile platform for depositing organic semiconductor layers, offering the advantages of large-area coverage and cost-effective fabrication. The utilization of solution processing has successfully yielded high-performance organic field-effect transistors (OFETs) [3,4], showing significant potential for applications in flexible logic circuits [5,6,7], sensors [8,9], nonvolatile memory [10], etc. For example, amplifier circuits featuring ultra-high voltage gain were achieved through the utilization of solution-processed monolayer organic crystals as channels [11].

However, due to the low viscosity of the solution, the crystallization of small-molecule semiconductors on the substrate often encounters de-wetting behavior, resulting in discontinuous and uneven thin films [12,13,14]. Therefore, it is still difficult to prepare large-area uniform and reproducible thin films based on small-molecule semiconductors. Polymers have high solution viscosity, good substrate wettability, and better uniformity of solution-processed thin films [15]. To improve the solution processability of small-molecule semiconductors, blending with polymers to form a vertical-phase-separation (VPS) structure has been demonstrated to be a successful route. A VPS structure will combine with the good electrical transport performance of small molecules and the good solution-processing properties of polymers, resulting in superior performance compared to single-component systems [4]. Liu et al. [16] found that the crystal size of pure 2,7-didodecyl[1]benzothieno[3,2-b][1]benzothiophene (C12-BTBT) thin films ranges from a few micrometers to several tens of micrometers, while the crystal size of thin films prepared by spin coating with a C12-BTBT/poly(methyl methacrylate) (PMMA)-blend solution is generally between several tens and several hundred micrometers, the crystal size significantly increases compared with pure C12-BTBT thin films. Hunter et al. [17] found that the increase in the polymer blend ratio during spin coating increased the coverage of the film. When the polymer addition was 50%, the 2,8-difluoro-5,11-bis(triethylsilylethynyl)anthradithiophene (diF-TESADT) exhibits complete crystallization in the upper layer of the film, not only improving the performance of the device but also improving the uniformity of the device due to the more uniform crystal surface of the film. Haase et al. [18] prepared 2,7-diocty[1]benzothieno[3,2-b]benzothiophene(C8-BTBT)/polystyrene (PS)-blend films using the meniscus shear method, and the film coverage gradually increased with the increase in PS fraction. The high fraction of insulating polymer further changes the morphology of C8-BTBT film, from highly arranged strips along the shear direction at a low fraction to adjacent strips perpendicular to the shear direction at 66% fraction. A thin film with good coverage and high crystal arrangement achieved an average migration rate of 14.4 cm^2^ V^−1^ s^−1^. In addition, due to the large area of high crystalline film formed by small-molecule semiconductors and the intrinsic semiconductor/dielectric layer interface, the influence of impurities and defects is reduced, which makes transistors have high device uniformity and electrical, environmental, and mechanical stability [2]. In order to regulate the crystallization of small molecules and prepare highly crystalline films, Zhang et al. [19] found that by blending polymers with small-molecule solutions, a viscosity gradient is formed on the meniscus during dip coating, promoting the transport of small molecules. In addition, polymer deposition at the bottom layer can also reduce the nucleation barrier of small molecules and improve the crystallinity of small-molecule films. They added PMMA with different blend ratios and molecular weights to α,ω-dihexylquaterthiophene (DH4T) solution and found that uniform strip film could be obtained under low pulling speed (5 μm s^−1^) and high blend ratio (10 wt%) conditions, while continuous DH4T films could be obtained under low pulling speed (5 μm s^−1^) and high PMMA molecular weight (2480 kDa) conditions only, exhibiting higher carrier mobility than other morphologies. In addition, high-performance field-effect transistors with a VPS structure can be realized by combining low-cost and large-area solution-processing technologies such as printing [20,21], meniscus shearing [22,23], and spray deposition [24,25].

Among the various solution-processing techniques, dip-coating exhibits the advantages of simplicity, efficiency, and the ability to generate highly oriented crystalline films with high performance [26,27]. During the dip-coating process, as a substrate is immersed into a solution, a meniscus is formed on the substrate due to capillary forces. This meniscus is stretched along the pulling direction by viscous forces, leading to the assembly of organic molecules along the three-phase contact line as the solvent evaporates. Consequently, a uniform organic crystalline film with oriented strips is formed, aligned along the pulling direction due to the relative movement between the substrate and the solution. Li et al. [28] have successfully fabricated isoindigo-based conjugated polymer films using dip-coating, exhibiting a field-effect mobility of up to 8.3 cm^2^ V^−1^ s^−1^ along the orientation direction. The performance is one order of magnitude higher than spin-coating transistors, attributable to enhanced polymer orientation. The morphology of dip-coating films can be influenced by factors, such as solution concentration, solvent evaporation rate, and pulling speed, etc. [29,30]. Zhao et al. and Wang et al. achieved aligned ribbon crystals by manipulating the evaporation rate through changes in the ratio of two solvents, inducing a stable Marangoni flow [31,32]. Additionally, highly crystalline organic films with self-aligned stripe patterns were obtained by adjusting parameters such as pulling speed and solution concentration [33]. Liu et al. proposed the meniscus angle as an index to quantify real-time and in-situ dynamic solution–substrate interactions during dip-coating, and a highly oriented C8-BTBT film was obtained through the proper surface treatment of the substrate at a low pulling rate [34].

Despite numerous attempts to optimize dip-coating techniques, there remain challenges in achieving continuous film morphology without gaps between strips. These gaps result in low coverage and a high number of traps at grain boundaries, hindering carrier transport. In this study, we addressed this issue by enhancing solution viscosity during dip-coating. The incorporation of a significant proportion of polymer binder into the solution led to the participation of more small molecules in deposition and the formation of a vertical-phase-separation film structure. OFETs based on these films exhibited a 100% increase in mobility compared to pure counterparts. This approach opens up possibilities for producing large-area and highly oriented continuous films, facilitating the development of high-performance OFETs.

## 2. Materials and Methods

### 2.1. Materials

In terms of materials, 2,6-Bis(4-octylphenyl)-dithieno[3,2b:2′,3′-d]thiophene (DTT-8, 99.9%) was purchased from Luminescence Technology Corp (Taiwan, China). and used as received without any further purification. Polymethyl methacrylate (PMMA, M_W_~350,000) was purchased from Sigma-Aldrich (Shanghai, China).

### 2.2. Device Fabrication

Transistors were fabricated on a highly n-doped silicon substrate with a 100 nm SiO_2_ layer as the gate electrode and dielectric layer, respectively. The substrates were cleaned by ultrasonic oscillation in deionized water, acetone, and ethyl alcohol for 20 min each, following UV irradiation treatment for 20 min. The solution was prepared in chlorobenzene by blending DTT-8 with the concentration of 5 mg mL^−1^ and PMMA with various ratios. Organic films were deposited by dip-coating with the hotpot temperature of 50 °C in a closed chamber to reduce interference, and without any post-treatment before OFETs construction. OFETs were constructed with a bottom-gate, top-contact (BGTC) configuration by thermal evaporation, with Ag as the source and drain electrodes. The channel length and width were 100 and 1000 μm, respectively.

### 2.3. Characterization

Electrical characteristics of the OFETs were measured with a semiconductor parameter analyzer (KEYSIGHT B2902A, Keysight Technologies, Santa Rosa, CA, USA) in a nitrogen atmosphere at room temperature. The field-effect mobility (*μ*) was calculated in the saturation regime by plotting the square root of the drain current versus the gate voltage using *I*_DS_ = (*W*/2*L*) *C_i_μ* (*V*_G_ − *V*_T_)^2^, where *I*_DS_ is the drain current, *C_i_* is the capacitance per unit area of the gate dielectric layer (*C_i_* = 30 nF cm^−2^), *V*_G_ is the gate voltage, *V*_T_ is the threshold voltage, and *W* and *L* are the channel width and length, respectively. The morphology of organic films was imaged by atomic force microscopy (AFM, Veeco Dimension 3100, Bruker, Billerica, MA, USA) in tapping mode. X-ray photoelectron spectroscopy (XPS) measurements were conducted on a Kratos Axis UltraDLD spectrometer (ESCALAB-250, ThermoFisher SCIENTIFIC, Waltham, MA, USA). The thin-film X-ray diffraction (XRD) pattern was recorded by an X-ray diffractometer (DX-2700B, HAOYUAN INSTRUMENT, Liaoning, China) with Cu Kα radiation. The static water contact angle was recorded and analyzed at room temperature by a video optical contact-angle measuring instrument (FM4200, KRÜSS GmbH, Shanghai, China).

## 3. Results and Discussion

DTT-8 was chosen as the model molecule due to its good solubility and excellent single-crystal mobility over 10 cm^2^ V^−1^ s^−1^ [35]. To enhance solution viscosity, PMMA, which is an insulating polymer with strong interaction with SiO_2_, good solubility, and low cost, was introduced into the DTT-8 solution, with chlorobenzene serving as the solvent.

**Figure 1:**
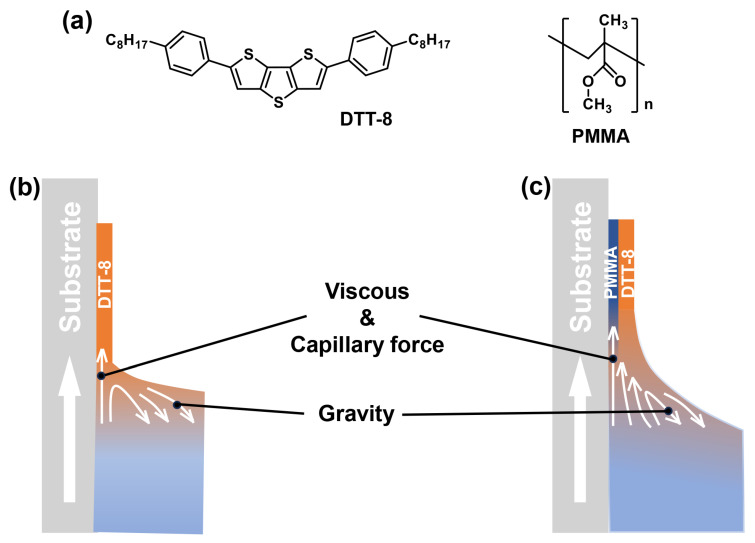
(**a**) Molecular structures of DTT-8 and PMMA. Schematic illustration of dip-coating process at the meniscus-controlled regime for pure DTT-8 solution (**b**) and the entrainment-controlled regime for DTT-8:PMMA blending solution (**c**).

**Figure 2:**
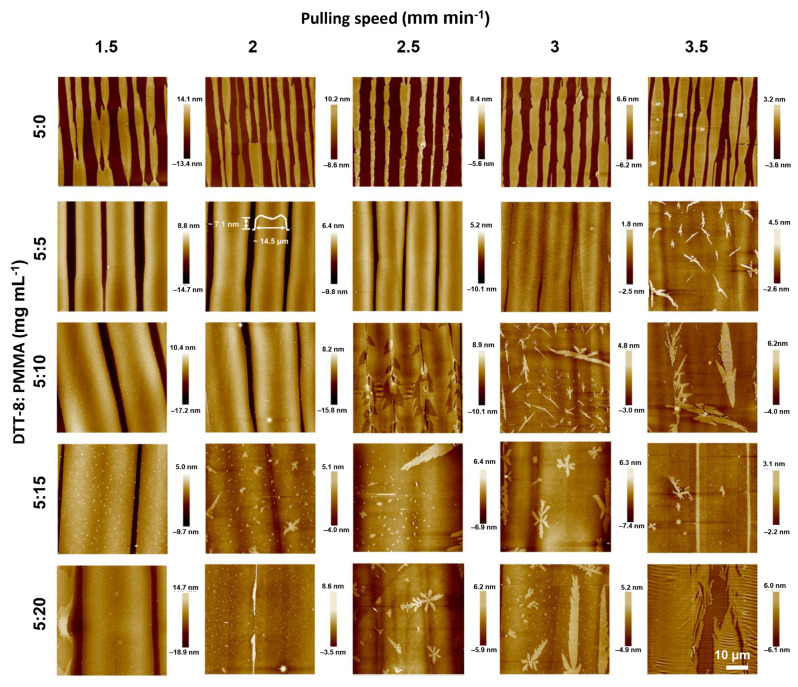
AFM images of dip-coated pure DTT-8 and DTT-8:PMMA films with varying pulling speeds and blend ratios. The scale of all AFM images is 50 × 50 μm^2^.

During the pulling process, a VPS structure was produced, which is investigated in the following text. The molecular structures of DTT-8 and PMMA and a schematic illustration of the dip-coating process are shown in Figure 1. Pure DTT-8 films without PMMA exhibit narrow strips with limited coverage, as shown in Figure 2. When the substrate is pulled up, an upward liquid flow emerges on the substrate surface due to the viscous force acting on the DTT-8 solution with constant capillary force and gravity. This flow continuously supplies solute, promoting nuclei and crystal growth at the three-phase contact line (TPCL) [31,32,35]. Consequently, oriented strips aligned with the pulling direction are produced. Upon increasing the pulling speed, the thicknesses of pure DTT-8 films decrease from approximately four monolayers (ML) to about one ML (Figure S1). This reduction indicates that film formation is under the meniscus-controlled regime [36,37,38] because of the low viscosity of the pure DTT-8 solution, as depicted in Figure 1b. The low viscosity constrains the mass transfer of organic molecules, leading to insufficient deposition and the formation of strips with noticeable gaps. Despite attempts to optimize pulling speed and solution concentration, these gaps are still hard to fill.

In order to provide more mass transfer during the pulling process, PMMA was introduced into the solution to enhance its viscosity. The blending of a polymer and small molecules is usually employed to solve the de-wetting problem of small molecules during solution processing [2,39]. As shown in Figure 2, the widths of the strips in the DTT-8:PMMA blend films surpass those of the pure DTT-8 films, and the corresponding gaps are gradually decreased with an increasing polymer blending ratio. At a modest blending ratio of 5:5 mg mL^−1^, the strips gradually widen with increasing pulling speed, maintaining a consistent thickness of ~2 ML. This observation implies that the film formation is governed by an entrainment-controlled regime (Figure 1c). The strip widths are more than 10 μm—much wider than those observed in pure DTT-8 films. As the blend ratios increase to 5:15 and 5:20 mg mL^−1^, the strips undergo further widening, and the gaps between strips are gradually filled, ultimately forming a highly oriented continuous film. A distinctive characteristic of this approach is the substantial increase in the quantity of polymers within the solution compared to conventional VPS structures [18,19]. When the blend ratio and pulling speed increase to a certain extent—for example, >2.5 mm min^−1^ and >5:10 mg mL^−1^, enough DTT-8 molecules form a continuous thin film, and the excess part of the DTT-8 molecules spontaneously forms flower-like/fractal-like crystals on the surface because they are not subjected to viscous force. This elevated polymer concentration significantly enhances the solution viscosity and the corresponding viscous force due to the entanglement of polymers within the blending solution. Consequently, the solution becomes entrained on the substrate, resulting in the retention of a substantial quantity of small molecules within the liquid film. This increased participation of organic molecules leads to the formation of oriented and continuous films.

**Figure 3:**
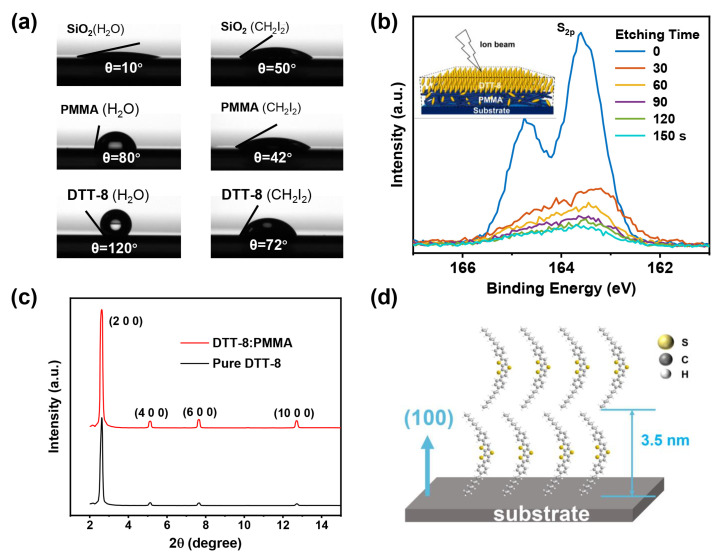
(**a**) Contact angles of water and CH_2_I_2_ on the surface of SiO_2_, PMMA, and DTT-8 thin films, (**b**) Evolution of S_2p_ peak intensity of XPS spectra with etching times, and the inset shows the VPS structure based on DTT-8 and PMMA on the substrate, (**c**) Out-of-plane XRD diffraction patterns of pure DTT-8 and DTT-8:PMMA films, (**d**) Schematic representation of DTT-8 molecules stacking on substrate.

A VPS structure manifests in the blend film of DTT-8 and PMMA, driven by their respective surface energies, which were determined by contact-angle measurements with water and diiodomethane, as illustrated in Figure 3a and detailed in Table 1. The SiO_2_ surface exhibits the highest surface energy, followed by PMMA and DTT-8. Consequently, DTT-8 molecules selectively accumulate at the uppermost surface of the film to minimize the overall energy, while PMMA resides at the film’s bottom. The establishment of this VPS structure is corroborated through further analysis. XPS in conjunction with ion-beam etching was utilized to investigate the vertical distribution of DTT-8, as depicted in Figure 3b. The S_2p_ peak, attributed to DTT-8, is notably more pronounced on the film surface. Subsequently, following 30 s of ion-beam etching, the intensity of the S_2p_ peak diminishes significantly, suggesting the accumulation of a substantial amount of DTT-8 on the film surface. With increasing etching time, the S_2p_ signal continues to attenuate, signifying a thorough phase separation between DTT-8 and PMMA. XPS analysis conclusively verifies the existence of a top layer of crystalline DTT-8 and a bottom layer of PMMA within the blend films.

**Table 1:** Contact angles and surface energy of water and diiodomethane on SiO_2_, PMMA, and DTT-8 surface.

Surface	Contact Angles (°)		$\gamma_{s}^{d}$ (mJ/m^2^)	$\gamma_{s}^{p}$ (mJ/m^2^)	$\gamma_{s}$ (mJ/m^2^)
	Water	Diiodomethane			
UV radiation SiO_2_	10	50	19.54	51.84	71.38
PMMA	80	42	34.81	4.45	39.26
DTT-8	130	72	26.11	2.31	28.42

The dispersion and polar components of surface energy, and the total surface energy was obtained from the following equation:

$$\left(1+cos\theta \right)\gamma_{l}=\gamma_{s}^{d}\;\gamma_{l}^{d}+\gamma_{s}^{p}{\;\gamma }_{l}^{p}$$

Among them, $\gamma_{s}$ and $\gamma_{l}$ stand for surface energy of films and liquids, respectively; *d* and *p* (in the superscript stand) for dispersion component and polar component, respectively; and $\gamma_{l}^{d}$ and $\gamma_{l}^{p}$ of water and diiodomethane are 48.5, 2.3 mJ/m^2^ and 51, 21.8 mJ/m^2^, respectively.

To investigate the packing structure of DTT-8 molecules on the polymer binder, XRD analysis was carried out, as shown in Figure 3c. The well-defined diffraction peaks of the (200), (400), (600), and (1000) planes [35] affirm that the orientation of the a-axis is perpendicular to the substrate, which also indicates that the π-π stacking interaction of DTT-8 molecules is parallel to the substrate [40,41] (Figure 3d). Noteworthy enhancements in peak intensities and reductions in half-peak widths in the blend film suggest an augmentation in crystallinity attributable to polymer blending, in comparison to the pure DTT-8 film. The increased involvement of molecules during film formation leads to wider strips and the development of a more continuous film. This observation underscores the positive impact of polymer blending on promoting improved packing of DTT-8 and enhanced crystallinity within the resulting film.

**Figure 4:**
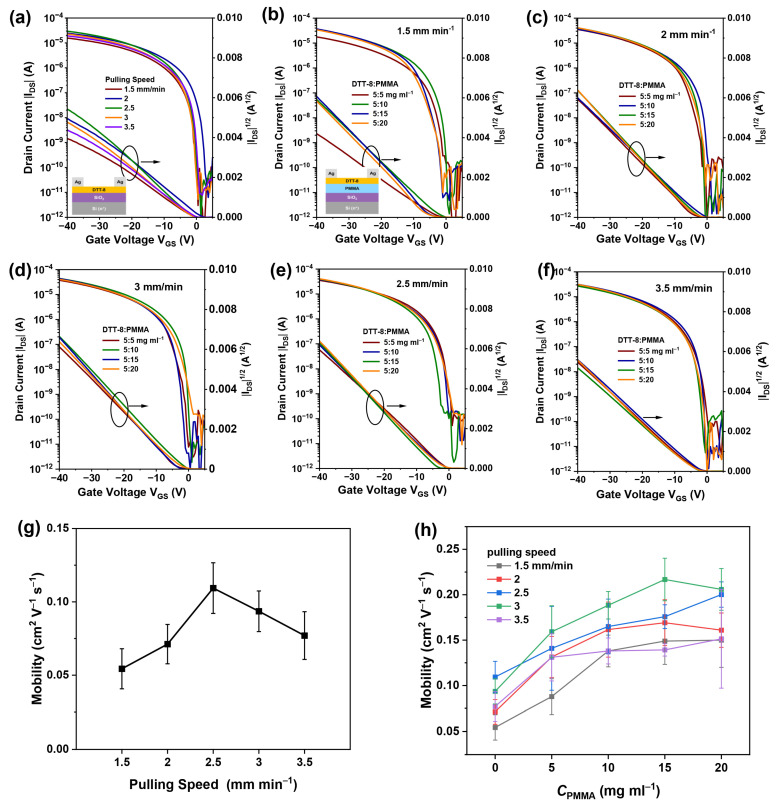
Transfer characteristics of pure DTT-8 transistors (**a**) with various pulling speeds and DTT-8:PMMA transistors at pulling speeds of 1.5 mm min^−1^ (**b**), 2 mm min^−1^ (**c**), 2.5 mm min^−1^ (**d**), 3 mm min^−1^ (**e**), 3.5 mm min^−1^ (**f**) with various blend ratios, (**g**) Pulling speed dependent saturation charge carrier mobility of pure DTT-8 transistors, (**h**) PMMA concentration dependent saturation charge carrier mobility of pure DTT-8 and DTT-8:PMMA transistors with different pulling speed.

To investigate charge transport within the highly oriented and continuous films, bottom-gate/top-contact OFETs were fabricated by employing DTT-8:PMMA VPS films as active layers, as shown in Figure 4. The transistor devices demonstrate typical hole accumulation operation mode. The saturation current of pure DTT-8 devices at a drain and gate voltage of −40 V increases first and then decreases with the pulling speed, reaching its maximum at a speed of 2.5 mm min^−1^, at which point the mobility reaches 0.11 cm^2^ V^−1^ s^−1^, as shown in Figure 4a,g. This is because the film thickness decreases with increasing pulling speed, and the coverage increases with increasing pulling speed. At low speeds, the film is thick enough, and thus, increasing coverage improves performance, while at high speeds, insufficient film thickness leads to a decrease in the accumulation of charge carriers in the channel, resulting in a decrease in performance. According to Figure 4b–f,h, DTT-8:PMMA transistor mobilities exhibit an incremental trend with an increasing pulling speed. For example, when the blend ratio is 5:15 mg mL^−1^, their hole mobilities show an increase from 0.14 cm^2^ V^−1^ s^−1^ in the 1.5 mm min^−1^ to 0.22 cm^2^ V^−1^ s^−1^ in the 3 mm min^−1^. This is due to the increase in film thickness with increasing pulling speed in the entrainment-controlled regime. However, it can be observed that at 3.5 mm min^−1^, the carrier mobility drops to 0.14 cm^2^ V^−1^ s^−1^, which also occurs in other blending ratios. This is because, at the speed of 3.5 mm min^−1^, randomly oriented crystal domains are formed in the film, reducing the orientation of the film and resulting in a decrease in device performance (but performance still far exceeds that of pure DTT-8 devices). At the same time, DTT-8:PMMA transistor mobilities also exhibit an incremental trend with an increasing PMMA ratio. For example, when the pulling speed is 3 mm min^−1^, their hole mobilities show an increase from 0.16 cm^2^ V^−1^ s^−1^ in the 5:5 mg mL^−1^ ratio to 0.22 cm^2^ V^−1^ s^−1^ in the 5:15 mg mL^−1^ ratio. This improvement can be attributed to the increased coverage and thickness of the VPS films. Although the performance of our OFETs does not reach the same level as the recently reported mobility of DTT-8-based OFETs [42,43], our method integrates the advantages of dip-coating, such as simplicity, speed, large-area fabrication, and high orientation. The realization of continuous films with elevated mobility substantiates that dip-coating utilizing high-viscosity solutions is an efficient method for depositing films of organic semiconductors, underscoring its potential in advancing the field of organic electronics.

**Figure 5:**
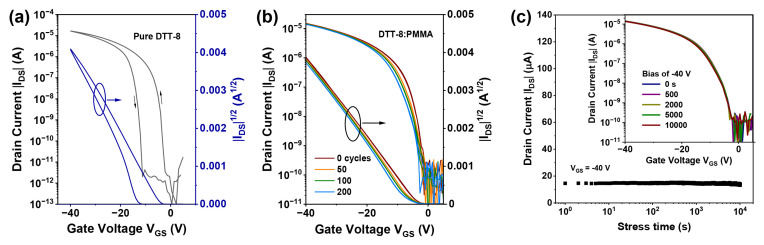
(**a**) Transfer characteristics of pure DTT-8 transistor, (**b**) Cycle stability of the DTT-8:PMMA transistor, (**c**) Bias stress of the channel current under continuous bias stress of −40 V for 10,000 s, and the inset shows the transfer curves taken before and after bias stress.

Hysteresis and bias stability serve as crucial metrics for evaluating the long-term performance of OFETs [44]. During cycling measurements, the transfer characteristics of pure DTT-8 film exhibit counterclockwise hysteresis with approximately −10 V, as depicted in Figure 5a. In contrast, the hysteresis of the transistor with a VPS structure is significantly reduced, with forward and backward characteristics nearly coinciding (Figure 5b). This device exhibits minimal hysteresis even after 200 cycles, with threshold voltages exhibiting slight fluctuations of about 0.3 V (Table 2). Further evaluation of the bias-stress stability of OFETs with a VPS structure is conducted at a drain and gate bias of −40 V, revealing that the drain current maintains its initial value of 15 μA for 10,000 s, as illustrated in Figure 5c. Their transfer curves indicate that the saturation current and threshold voltage show negligible changes after continuous bias for 500, 2000, 5000, and 10,000 s. The observed minimal hysteresis and high bias stability can be attributed to the VPS structure, which significantly increases the interface quality between the semiconductor and dielectric layer [45,46]. PMMA at the bottom layer passivates interface traps arising from polar hydroxyl groups on the SiO_2_ surface [46,47]. Furthermore, the oriented and continuous film eliminates gaps between strips and traps at grain boundaries, which further improves film crystallinity. Consequently, the resulting OFETs exhibit many advantages, including an absence of hysteresis, excellent bias stability, low threshold voltage, and high carrier mobility.

## 4. Conclusions

In this study, we have developed a novel approach for the fabrication of highly oriented and continuous films, leveraging the synergy of dip-coating technology and a vertical-phase-separation structure. The enhanced viscosity of the solution facilitates the mass transfer of organic molecules during the dip-coating process, which leads to the participation of more molecules in the formation of organic films, thereby eliminating gaps between oriented strips. Moreover, the integration of the VPS structure contributes to an enhanced quality of the intrinsic semiconductor/dielectric interface. A comprehensive exploration of the correlation between film morphology and the polymer ratio was conducted, revealing that the addition of a substantial proportion of PMMA into the solution led to the attainment of high-crystalline DTT-8 films with oriented strips and an absence of gaps. As a result, the fabricated OFETs showed improved mobility and operationally stable features, and they are also hysteresis-free. Our method offers an effective solution for the growth of highly oriented and continuous films, contributing significantly to the cost-effective and large-scale production of organic transistors.

## Figures and Tables

**Table 2:** Transistor performance of DTT-8:PMMA VPS film by cycling measurements.

Cycles	Forward		Backward		Δ*μ*	Δ*V_T_*
	*μ* (cm^2^ V^−1^ s^−1^)	*V_T_* (V)	*μ* (cm^2^ V^−1^ s^−1^)	*V_T_* (V)		
initial	0.074	3.00	0.076	3.28	0.002	0.27
20	0.078	3.92	0.083	4.31	0.006	0.39
40	0.079	4.31	0.083	4.54	0.004	0.23
60	0.080	4.61	0.084	4.85	0.004	0.24
80	0.081	4.86	0.085	5.12	0.005	0.26
100	0.081	5.05	0.084	5.23	0.003	0.18
120	0.081	5.20	0.088	5.67	0.007	0.47
140	0.082	5.37	0.088	5.81	0.006	0.44
160	0.082	5.58	0.089	6.05	0.007	0.47
180	0.083	5.89	0.090	6.23	0.007	0.34
200	0.093	6.62	0.091	6.42	−0.002	−0.20

## Data Availability

The original contributions presented in the study are included in the article; further inquiries can be directed to the corresponding authors.

## Supplementary Materials

The following supporting information can be downloaded at: https://www.mdpi.com/article/10.3390/ma17071465/s1, Figure S1: AFM images and height profiles of dip-coated DTT-8 films by varying the pulling speed with the solution concentration of 5 mg mL^−1^. Thickness of DTT-8 monolayer film is about 3.5 nm. With increasing the pulling speed, the thicknesses of DTT-8 strips are reduced from 4 ML of 1.5 mm min^−1^ to 1 ML of 3.5 mm min^−1^.
