# Supplementary material for: Hysteresis-Free and Bias-Stable Organic Transistors Fabricated by Dip-Coating with a Vertical-Phase-Separation Structure
Source: Materials (Basel). 2024 Mar 22;17(7):1465. doi: 10.3390/ma17071465 (PMC11012522; doi:10.3390/ma17071465)
Supplement: Supplementary file 1 [file materials-17-01465-s001.zip › materials-2893453-supplementary.pdf]

Supplementary Materials

Hysteresis-Free and Bias-Stable Organic Transistors with Oriented and Continuous Films Fabricated through Dip-Coating with a Vertical Phase Separation Structure

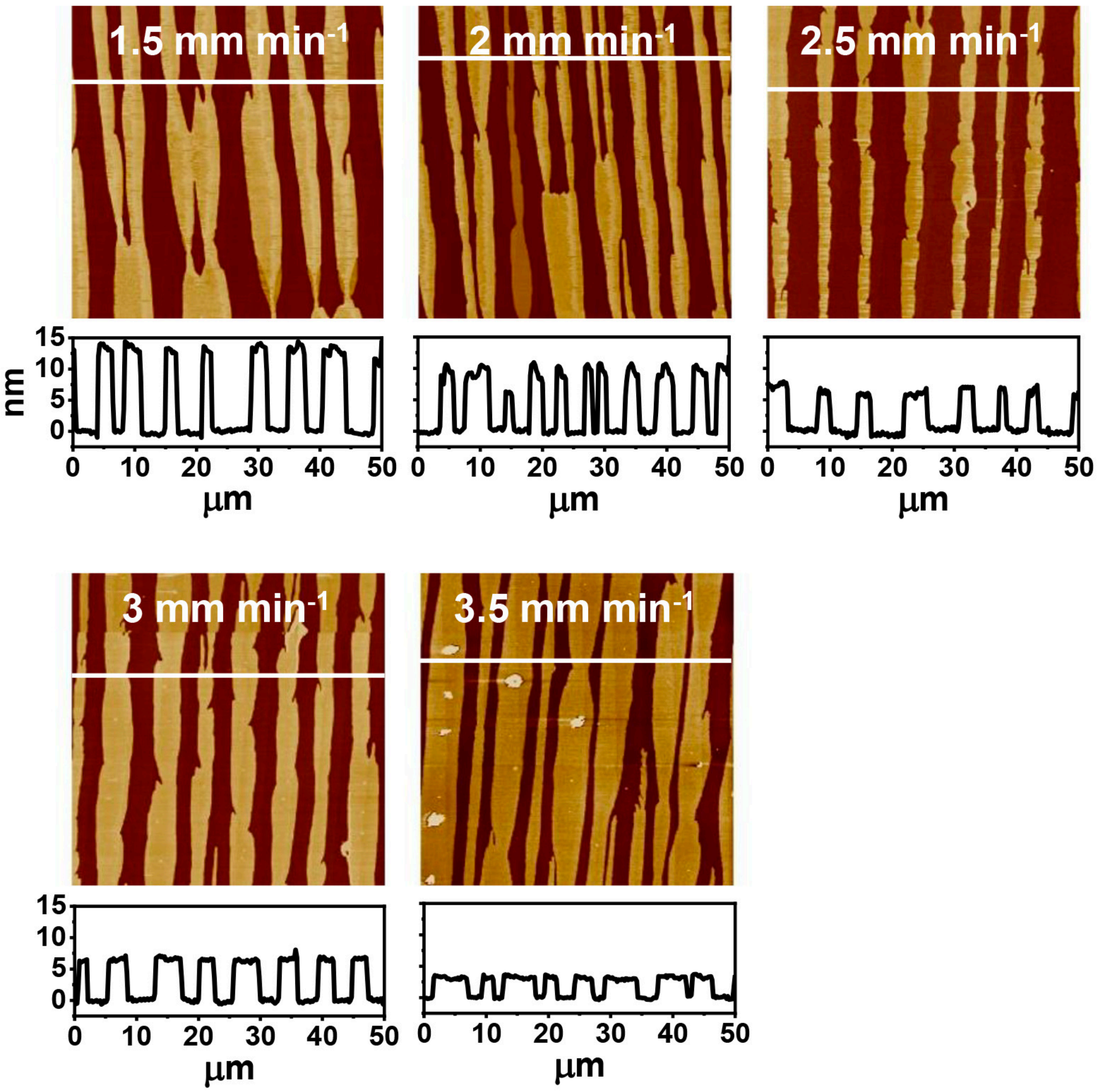

Figure S1. AFM images and height profiles of dip-coated DTT-8 films by varying the pulling speed with the solution concentration of 5 mg mL-1. Thickness of DTT-8 monolayer film is about 3.5 nm. With increasing the pulling speed, the thicknesses of DTT-8 strips are reduced from 4 ML of 1.5 mm min-1 to 1 ML of 3.5 mm min-1.
